# Supplementary material for: Reconstructing the Backbone of the Saccharomycotina Yeast Phylogeny Using Genome-Scale Data
Source: G3 (Bethesda). 2016 Sep 26;6(12):3927–39. doi: 10.1534/g3.116.034744 (PMC5144963; doi:10.1534/g3.116.034744)
Supplement: Supplemental Material [file supp_6_12_3927__index.html]

Reconstructing the Backbone of the Saccharomycotina Yeast Phylogeny Using Genome-Scale Data — Reconstructing the Backbone of the Saccharomycotina Yeast Phylogeny Using Genome-Scale Data — Supplemental Material 

# Reconstructing the Backbone of the Saccharomycotina Yeast Phylogeny Using Genome-Scale Data

## Supplemental Material for Shen, *et al*, 2016

**Files in this Data Supplement:**

- Figure S1 - Box plots of GC content for the 1,233 nuclear protein-coding BUSCO genes in each of the 96 taxa used in this study. (.pdf, 3,849 KB)
- Figure S2 - Distribution of the best-fitting amino acid substitution models across the amino acid alignments of the 1,233 BUSCO genes used in this study. (.pdf, 253 KB)
- Figure S3 - Venn diagrams illustrating the overlaps between subsets (left, Top 25%; right, Top 50%) of 1,233 genes displaying the highest average bootstrap support (ABS) and the highest average relative tree certainty (RTC) in their ML gene trees. (.pdf, 560 KB)
- Figure S4 - Illustration of occupancy for each taxon and gene in our data matrices. Each row corresponds to a taxon and each column corresponds to a BUSCO gene. (.pdf, 2,880 KB)
- Figure S5 - Distribution of gene alignment lengths across the 1,233 BUSCO genes used in this study. (.pdf, 296 KB)
- Figure S6 - The phylogenetic relationships of Saccharomycotina yeasts inferred from the concatenation-based analysis of the C12 data matrix. (.pdf, 314 KB)
- Figure S7 - The phylogenetic relationships of Saccharomycotina yeasts inferred from the coalescence-based analysis of the C12 data matrix. (.pdf, 310 KB)
- Figure S8 - Phylogenetic supernetwork of Saccharomycotina yeasts inferred from the 1,233 partial gene trees estimated from the AA data matrix (a) and the C12 data matrix (b). (.pdf, 674 KB)
- Figure S9 - Conflicts in the phylogenetic relationships of Saccharomycotina yeasts inferred from the concatenation-based (a) and coalescence-based (b) analysis of the 308 genes in the AA data matrix whose bootstrap consensus gene trees had the highest average bootstrap support (top 25%). (.pdf, 350 KB)
- Table S1 - List of all taxa used in this study. (.xlsx, 23 KB)
- Table S2 - Summary of assessment of 96 genome assemblies. (.xlsx, 15 KB)
- Table S3 - Summary of 1,233-gene, 96-taxon data matrix. (.xlsx, 40 KB)
- Table S4 - GO term enrichment analysis of 1,437 BUSCO genes. (.xlsx, 20 KB)
