# Supplementary figures and images for: Reconstructing the Backbone of the Saccharomycotina Yeast Phylogeny Using Genome-Scale Data
Source: G3 (Bethesda). 2016 Sep 26;6(12):3927–39. doi: 10.1534/g3.116.034744 (PMC5144963; doi:10.1534/g3.116.034744)

Figure S1

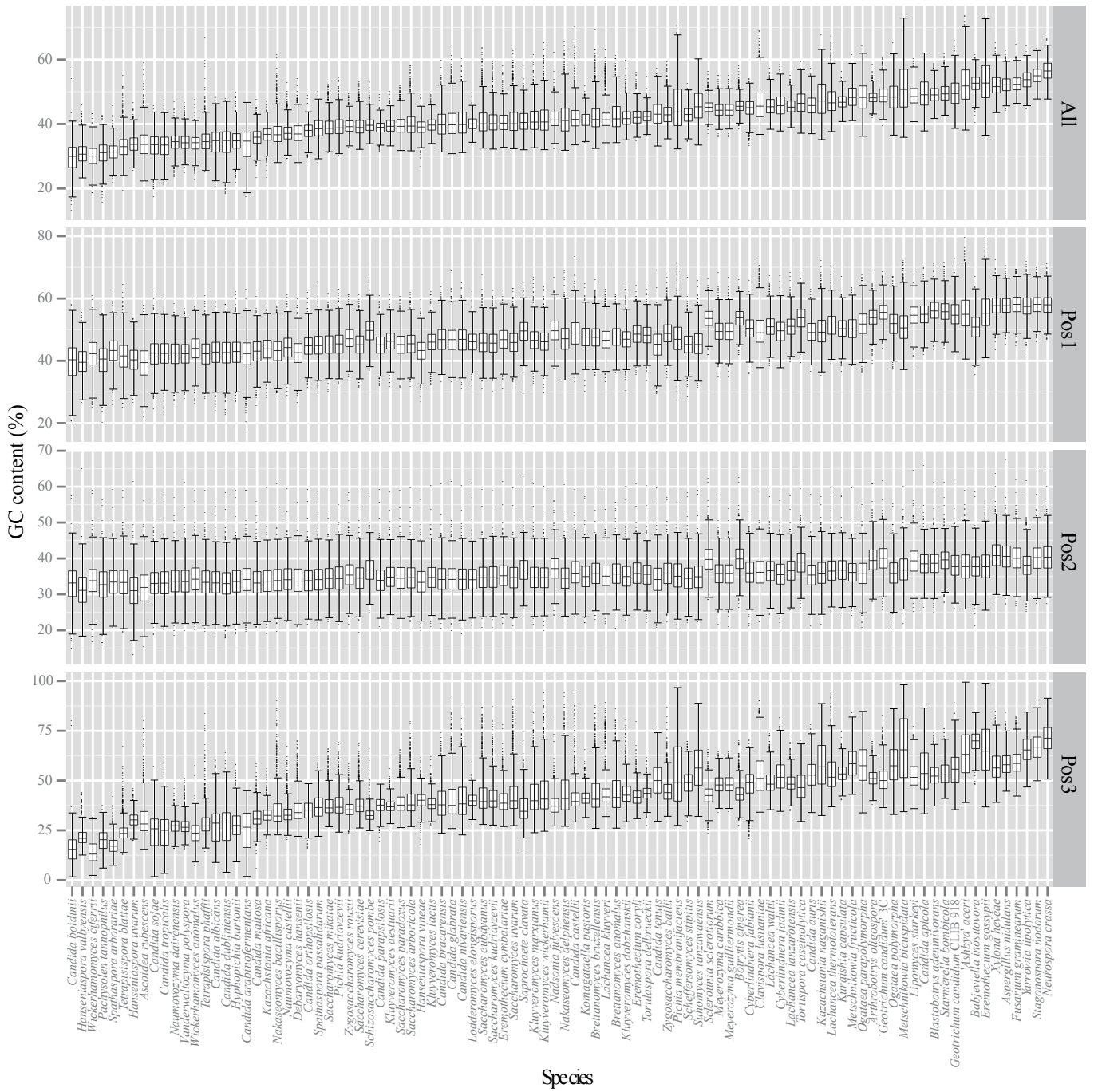

Supplement: Supplemental Material [file supp_g3.116.034744_FigureS1.pdf]

Figure S2

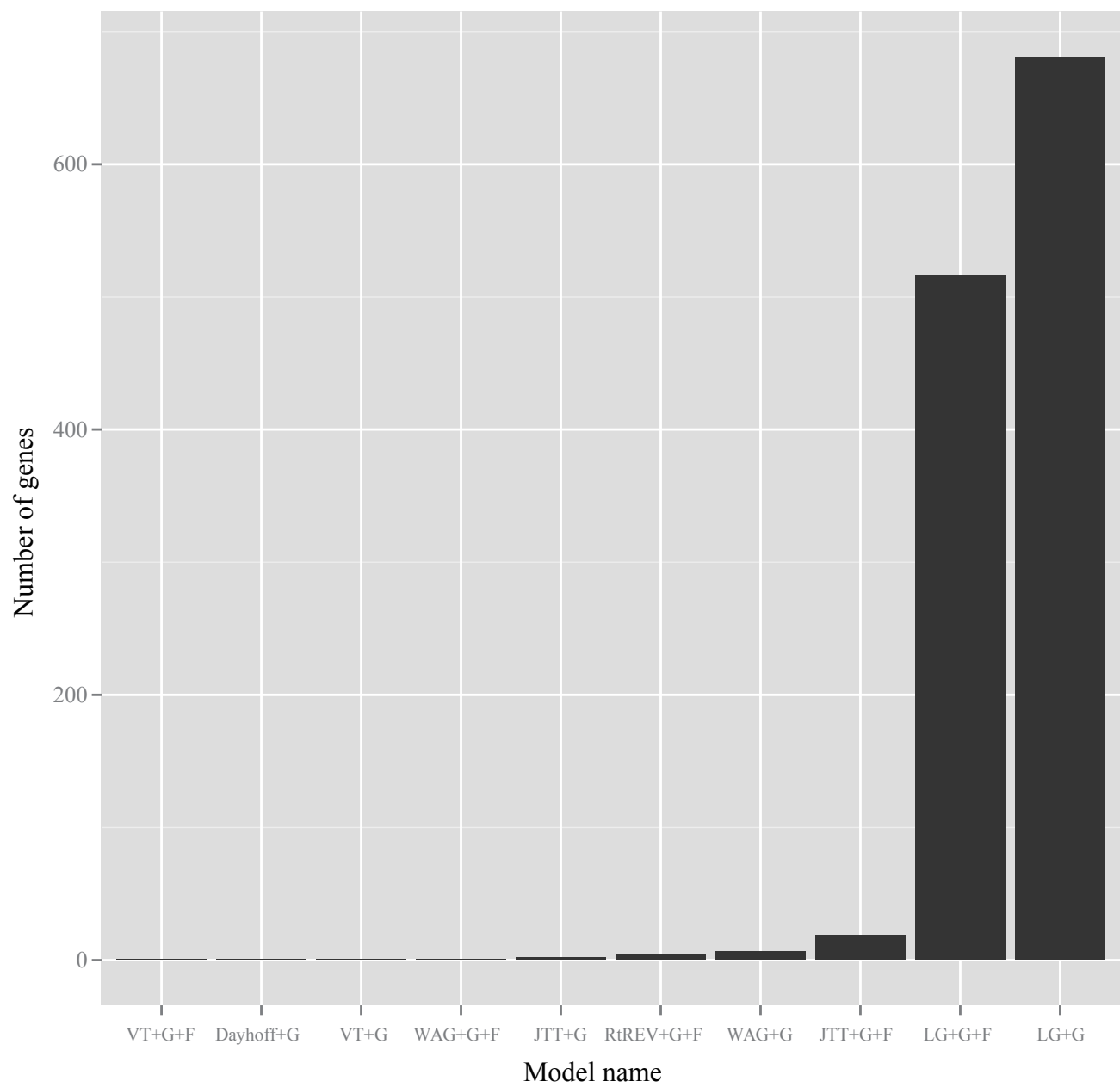

Supplement: Supplemental Material [file supp_g3.116.034744_FigureS2.pdf]

Figure S3

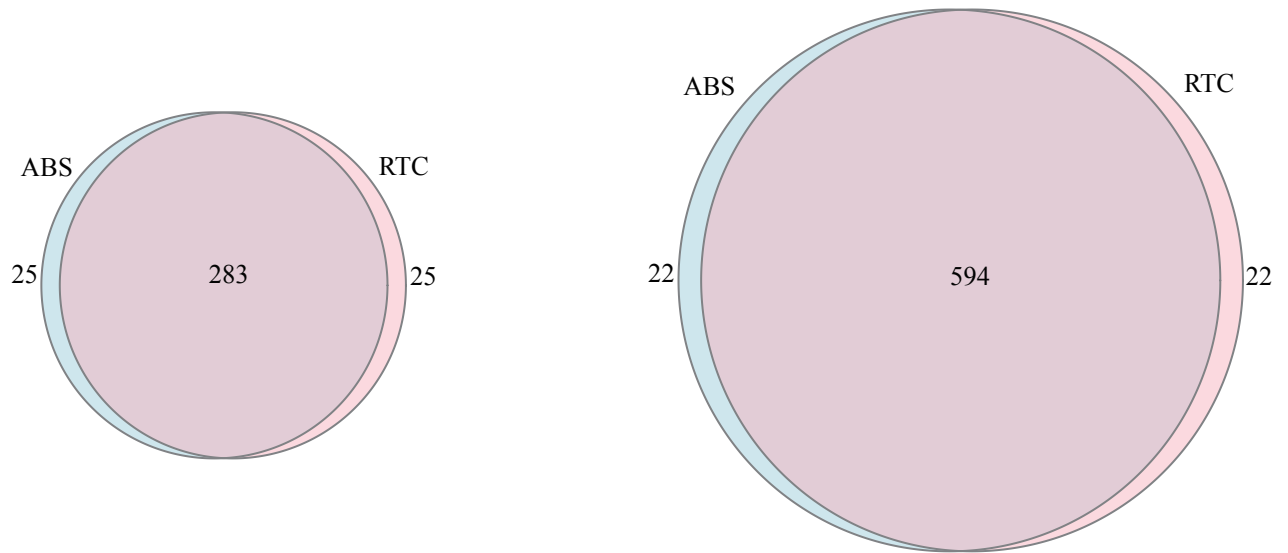

Supplement: Supplemental Material [file supp_g3.116.034744_FigureS3.pdf]

Figure S4

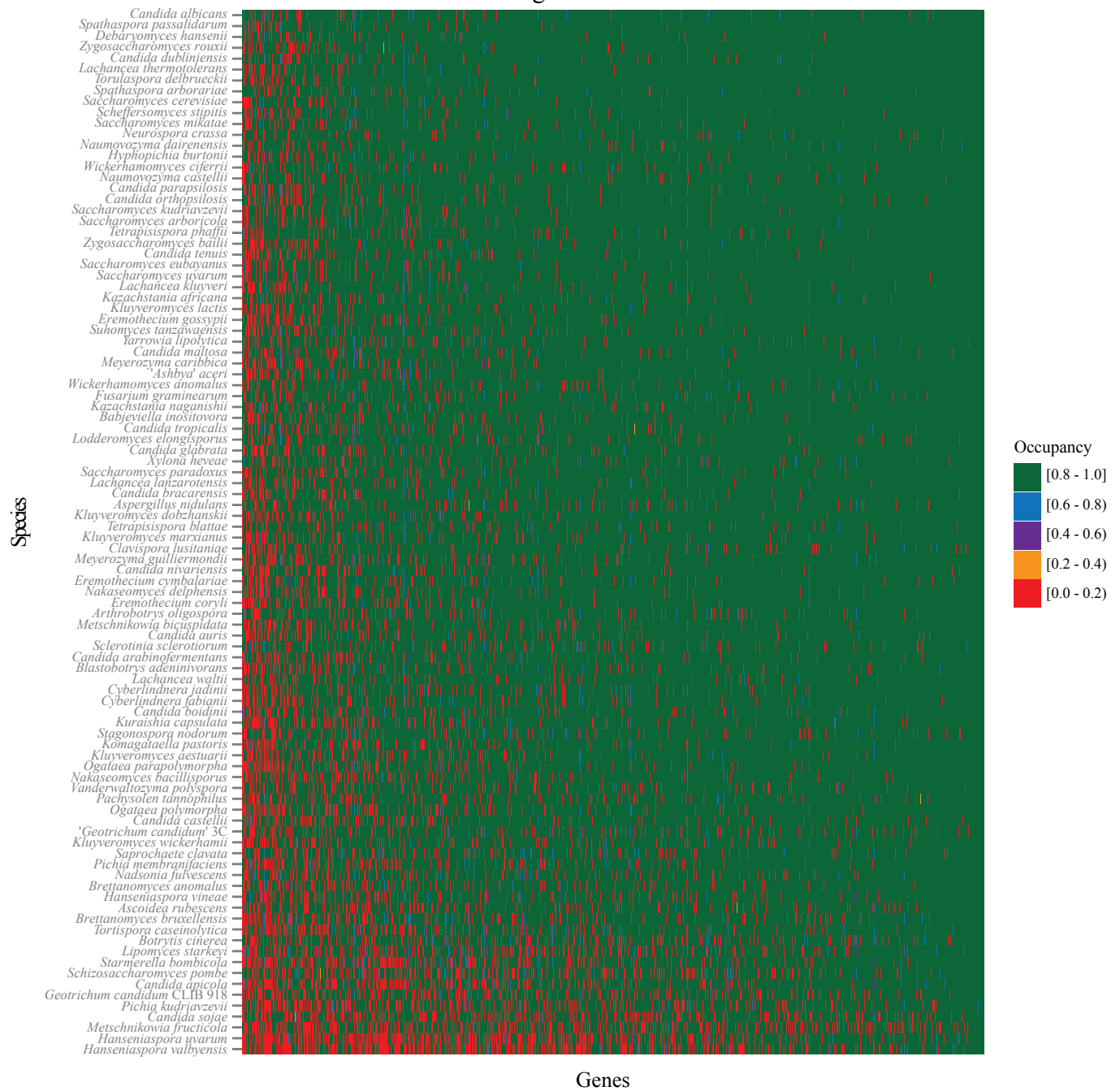

Supplement: Supplemental Material [file supp_g3.116.034744_FigureS4.pdf]

Figure S5

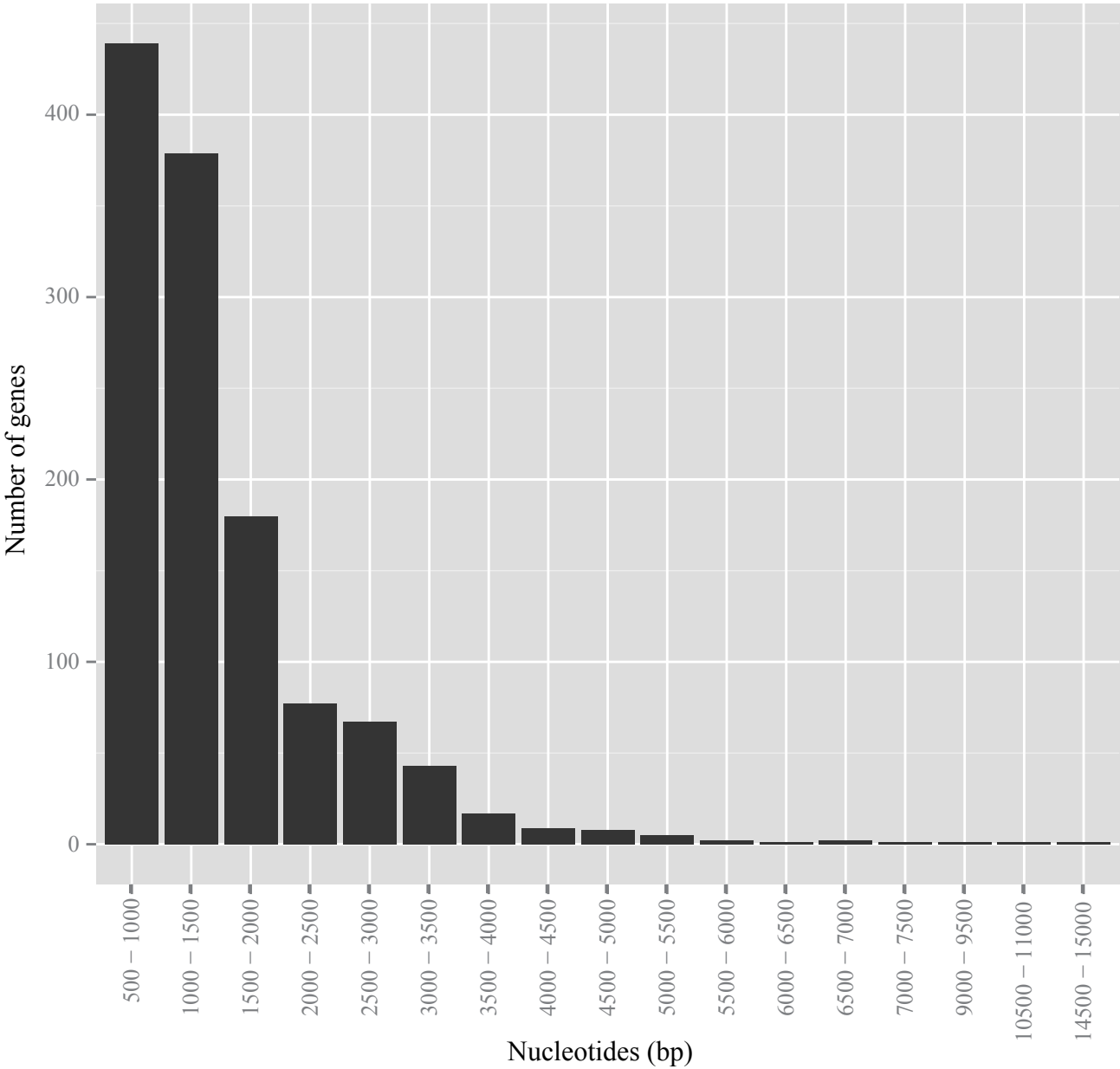

Supplement: Supplemental Material [file supp_g3.116.034744_FigureS5.pdf]

Figure S6

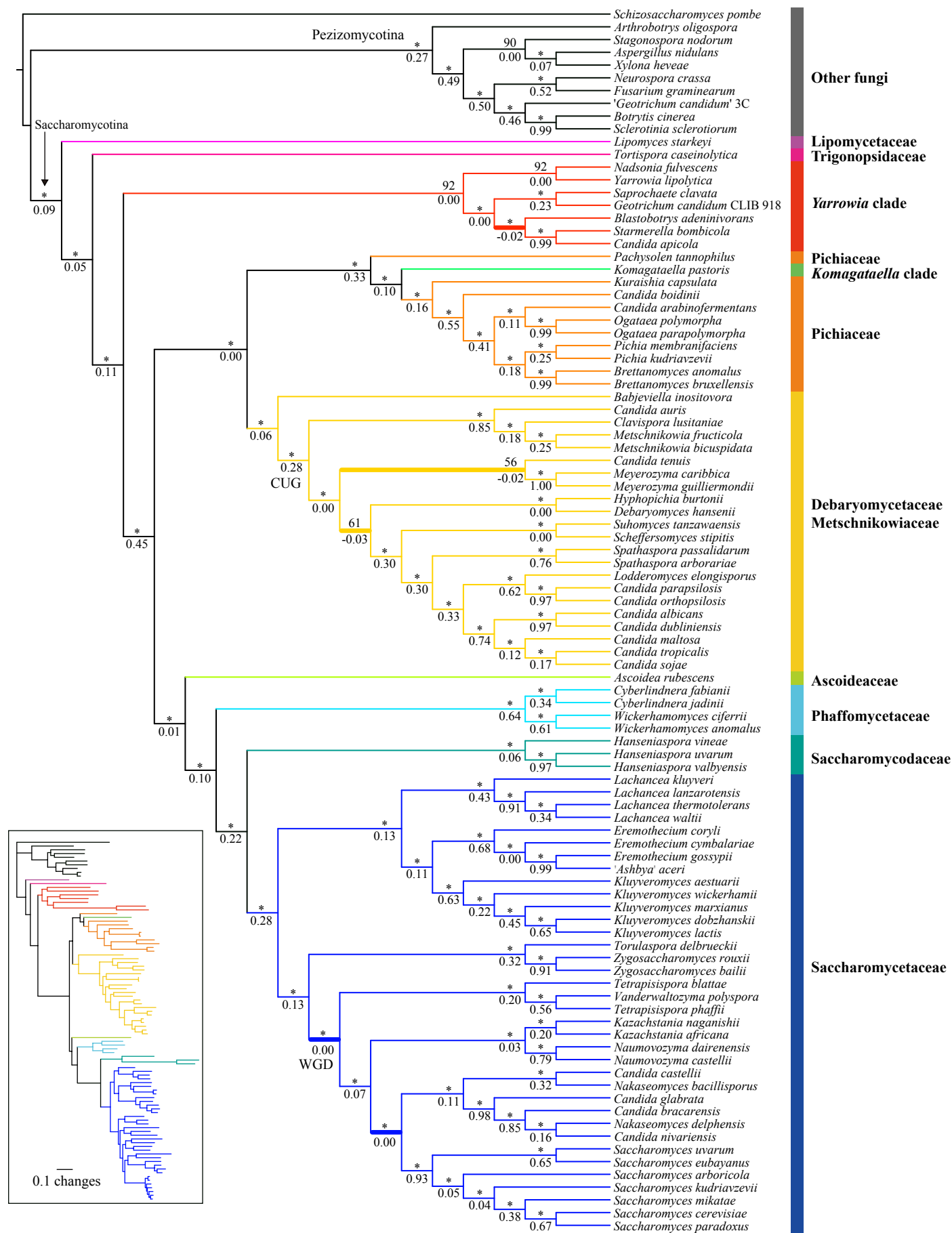

Supplement: Supplemental Material [file supp_g3.116.034744_FigureS6.pdf]

Figure S7

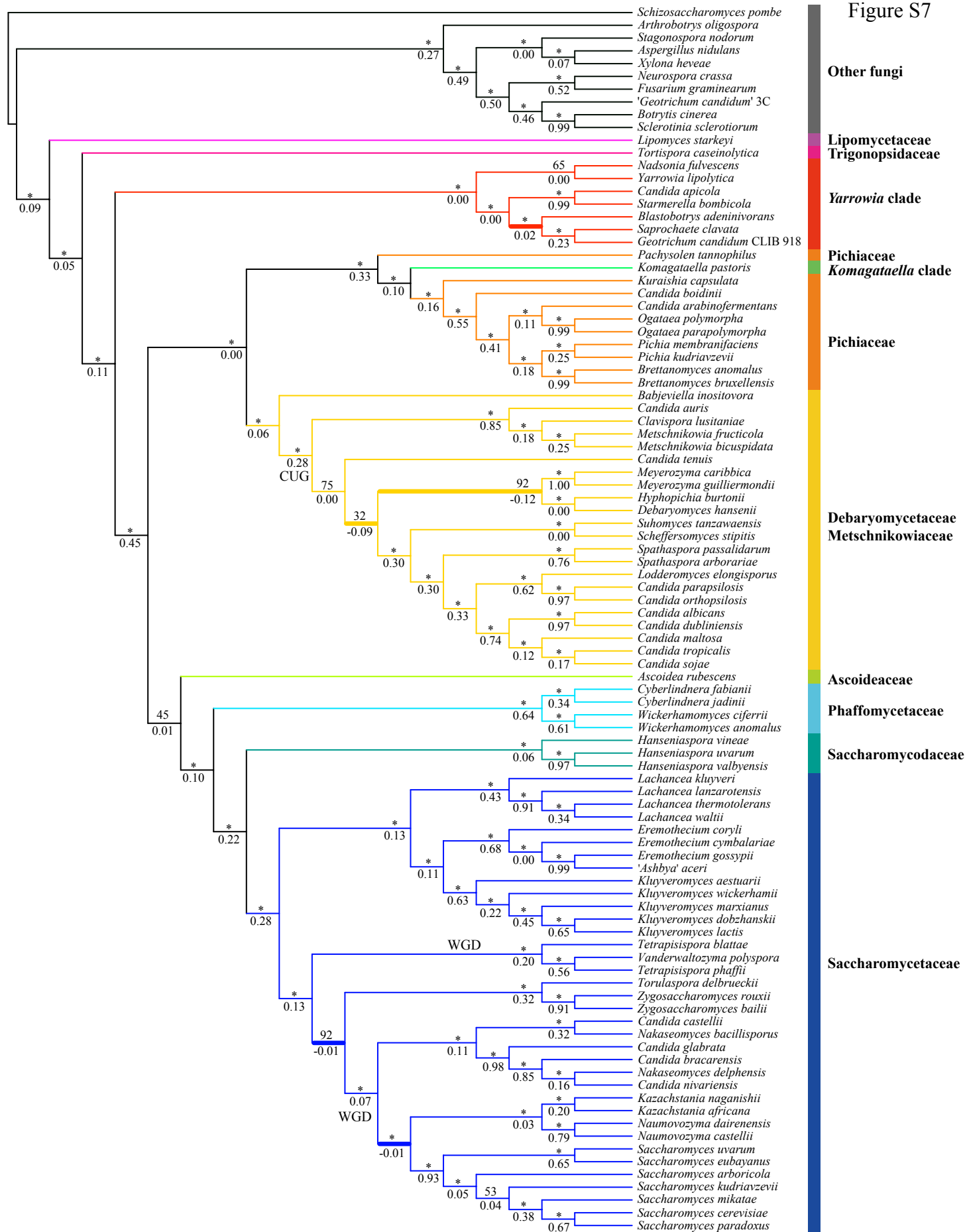

Supplement: Supplemental Material [file supp_g3.116.034744_FigureS7.pdf]

Figure S8

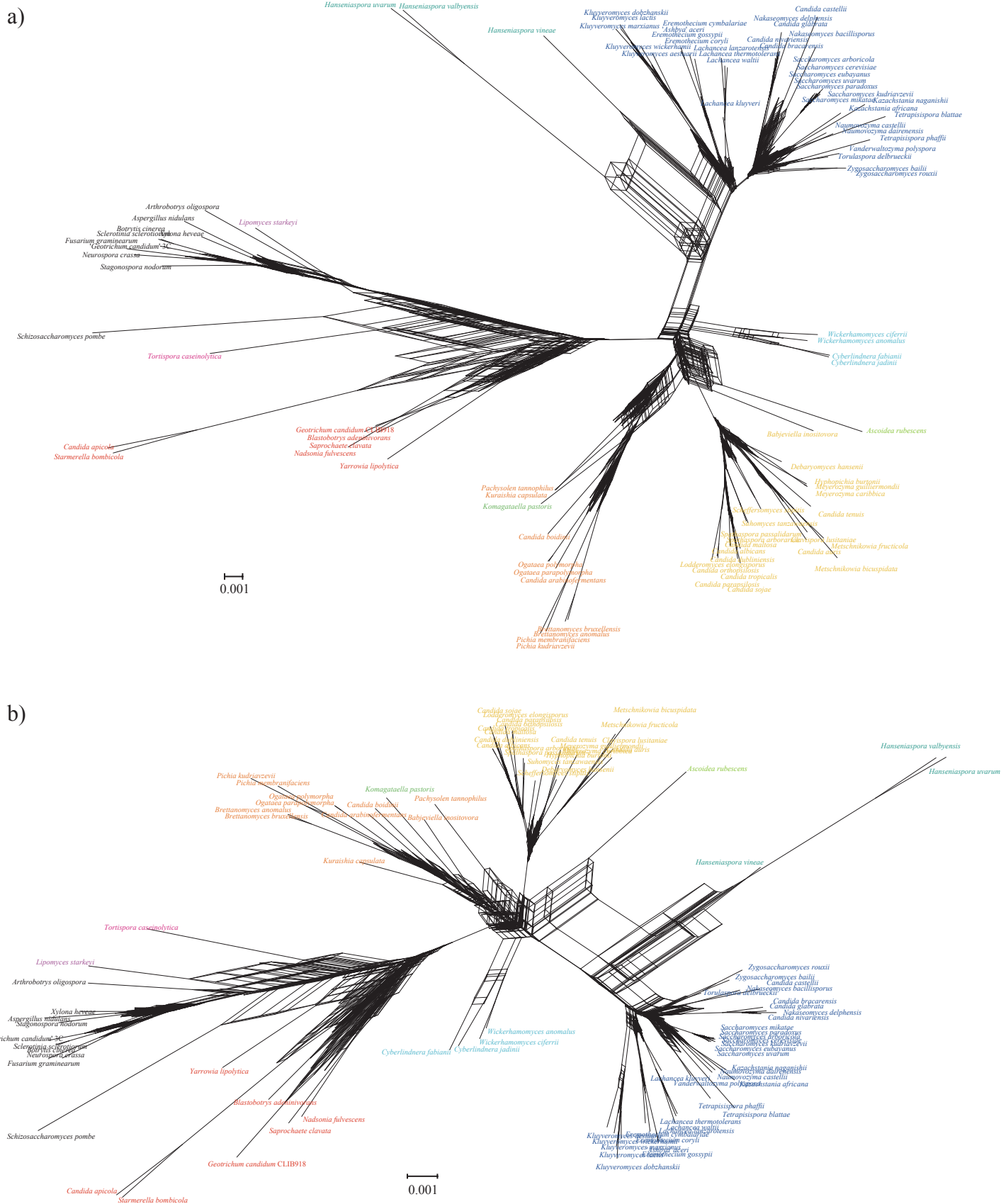

Supplement: Supplemental Material [file supp_g3.116.034744_FigureS8.pdf]

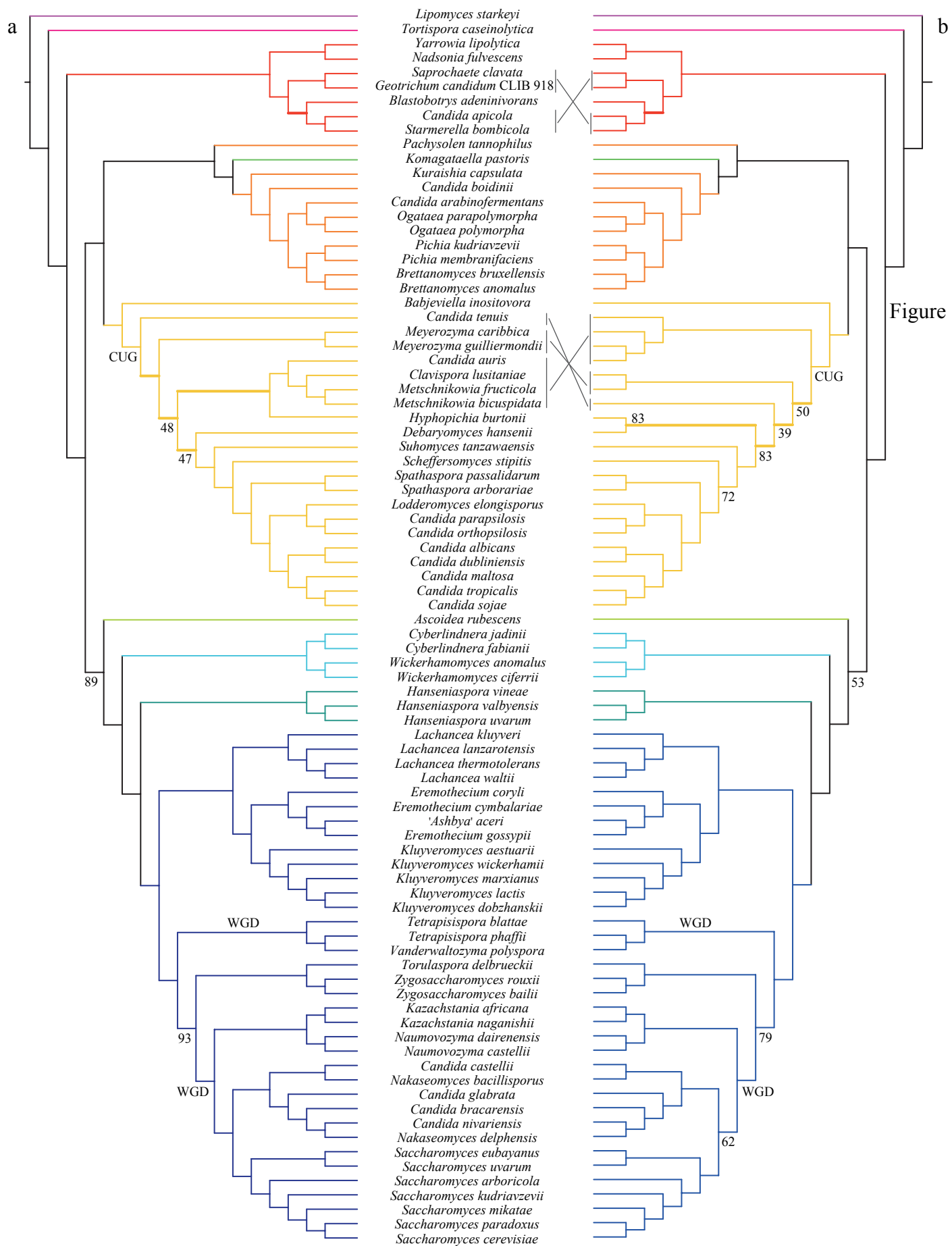

Supplement: Supplemental Material [file supp_g3.116.034744_FigureS9.pdf]
